# A threefold rise in widespread extreme rain events over central India

**DOI:** 10.1038/s41467-017-00744-9

**Published:** 2017-10-03

**Authors:** M. K. Roxy, Subimal Ghosh, Amey Pathak, R. Athulya, Milind Mujumdar, Raghu Murtugudde, Pascal Terray, M. Rajeevan

**Affiliations:** 1 Centre for Climate Change Research, Indian Institute of Tropical Meteorology, Pune, 411008 India; 2 Department of Civil Engineering, Indian Institute of Technology Bombay, Mumbai, 400076 India; 3 Department of Physical Oceanography, Cochin University of Science and Technology, Kochi, 682016 India; 4 Earth System Science Interdisciplinary Center, University of Maryland, College Park, MD 20742 USA; 5 Sorbonne Universites (UPMC, Univ. Paris 06)-CNRS-IRD-MNHN, LOCEAN Laboratory, 4 Place Jussieu, F-75005 Paris, France; 6 Indo-French Cell for Water Sciences, IISc-IITM-NIO–IRD Joint International Laboratory, IITM, Pune, 411008 India; 7 Ministry of Earth Sciences, Government of India, Lodhi Road, New Delhi, 110003 India

## Abstract

Socioeconomic challenges continue to mount for half a billion residents of central India because of a decline in the total rainfall and a concurrent rise in the magnitude and frequency of extreme rainfall events. Alongside a weakening monsoon circulation, the locally available moisture and the frequency of moisture-laden depressions from the Bay of Bengal have also declined. Here we show that despite these negative trends, there is a threefold increase in widespread extreme rain events over central India during 1950–2015. The rise in these events is due to an increasing variability of the low-level monsoon westerlies over the Arabian Sea, driving surges of moisture supply, leading to extreme rainfall episodes across the entire central subcontinent. The homogeneity of these severe weather events and their association with the ocean temperatures underscores the potential predictability of these events by two-to-three weeks, which offers hope in mitigating their catastrophic impact on life, agriculture and property.

## Abstract (web summary)

Against the backdrop of a declining monsoon, the number of extreme rain events is on the rise over central India. Here the authors identify a threefold increase in widespread extreme rains over the region during 1950–2015, driven by an increasing variability of the low-level westerlies over the Arabian Sea.

## Introduction

Global economic losses from floods exceeded $30 billion per year in the past decade, with some of the largest losses linked to extreme rainfall events in Asia (International Disaster Data Base, http://www.emdat.be). Floods attributed to extreme rain events in India alone amounted to losses of about $3 billion per year, which is 10% of the global economic losses. The plains of central India are largely flood-prone; flash floods, landslides and torrential rains often kill thousands and displace millions of people as well as animals, underscoring the urgency in comprehending and predicting these events. There have been 268 reported flooding events in India over 1950–2015 affecting about 825 million people, leaving 17 million homeless and killing 69,000 people (International Disaster Data Base). Many of these events which caused large loss of life, property and agriculture^1^ occurred across central India.

**Fig. 1:**
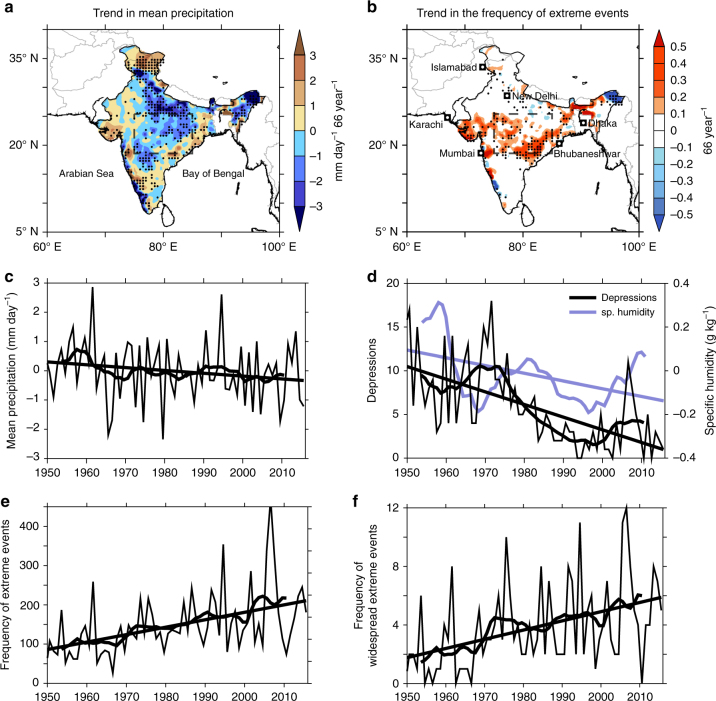
Trends in summer mean and extreme precipitation during 1950–2015. Observed trend in summer **a** mean precipitation anomalies (mm day^−1^ 66 year^−1^) and **b** the frequency (66 year^−1^) of extreme precipitation events (precipitation ≥ 150 mm day^−1^). Mean precipitation for the season is 8.1 mm day^−1^. Time series of **c** of precipitation (mm day^−1^), **d** specific humidity (1000–200 hPa) anomalies (g kg^−1^), and the number of days with low-pressure systems over central India and **e** frequency of extreme rain events (number of grid cells exceeding 150 mm day^−1^ per year) over central Indian subcontinent (75°–85° E, 19°–26° N, inset boxes in **a**, **d**). **f** Time series of the frequency of widespread extreme events (number of days when the extreme events simultaneously cover ten grid cells or more). Stippling indicates trend values significant at 95% confidence level. The trend lines shown in the figures are significant at 95% confidence level. The smoothed curves on the time series analyses represent 10-year moving averages. The entire analysis is for the northern summer (June-September), for the years 1950–2015. The precipitation and cyclone data is based on IMD observations, and the specific humidity is based on NCEP reanalysis. See the “Methods” section for more information regarding the data

The variability of the monsoon makes the heavily populated South Asian subcontinent one of the most vulnerable regions around the world to the impacts of natural disasters such as droughts and floods. The monsoon variability has amplified in the recent decades^2–8^, with a gradual decline in the monsoon circulation and rainfall^9–13^ and at the same time, a phenomenal rise in extreme rainfall events^2, 5, 14–20^. Observational evidence indicates a decrease in the northern summer (June–September) mean rainfall over South Asia since the 1950s, with a significant decline of 10–20% over the central Indian region where agriculture is still largely rain-fed (Fig. 1a). The consistent decline in the mean rainfall is attributed to a weakening monsoon circulation, owing to a combination of factors including the warming of the Indian Ocean^11–13^, increasing frequency and magnitude of El Niño events^21, 22^, increased air pollution^16^ and land use changes^16, 23^ over the subcontinent. Meanwhile, the extreme rainfall events in the subcontinent are on the rise^14–16, 19^ (Fig. 1b), with up to a 10–30% increase over the central Indian subcontinent^16^. Furthermore, several analysis suggest that while the overall intensity and frequency of extreme events are increasing over the region, at local scale they are spatially non-uniform with increasing spatial variability^5, 24–27^. With the projected changes showing further intensification of extreme precipitation over most parts of the subcontinent by the end of the century^28^, the need for rapid progress on process and predictive understanding of these extremes can hardly be overemphasized.

Despite an understanding of the past and projected increase in the number of extreme rainfall events, the suggested links between the increasing events and the warming environment remain contested^14–16, 29^. A few studies^14, 15^ suggest that the rise in extreme rainfall events over central India is due to an increase in the moisture content, which they link to the rapid warming of the equatorial Indian Ocean^14, 15, 20, 30^. Other studies^16, 20^ propose that the local surface warming over the Indian subcontinent and the corresponding rise in humidity levels have a role in the increasing frequency of events. It is generally presumed that many of these heavy rainfall spells result from low-pressure systems (depressions and cyclones with a wind speed ≥ 8.5 m s^−1^) that develop in the Bay of Bengal, with typical time scales of 3–5 days, and move northwestward bringing moisture into the central Indian subcontinent^18, 31–36^. Climate model simulations^33, 37^ suggest an increase in these low-pressure systems, bringing in more moisture in a warmer environment. On the contrary, observations indicate that the frequency of these depressions and cyclones has in fact declined^33, 38–40^, raising some critical questions. How is the increase in the frequency of extreme events sustained despite a weakened monsoon circulation and a decrease in the number of depressions over central India? Is the moisture content increasing despite a declining monsoon, and if so, what ensures the availability of moisture to foster these extreme events? Is it that the depressions are bringing in more moisture even though their frequency has declined? It is well known that the low-level westerlies play a major role in contributing to the mean monsoon rainfall during summer^41–44^ and also supply moisture for the depressions forming over the Bay of Bengal^31, 39^. However, the monsoon southwesterlies over South Asia are weakening in the recent decades^12, 16, 39^, raising further questions on the mechanisms driving the increased extreme rain events.

In this study, we find that there is a threefold increase in widespread extreme precipitation events over central India during 1950–2015. Our results reveal that moisture transport from the Arabian Sea is the major contributor to the buildup of these events. We find that the low-level monsoon westerlies over the northern Arabian Sea exhibit increasing variability, driving surges of moisture supply, leading to extreme rainfall episodes across the entire central subcontinent. The increasing variability of these westerlies appears to be linked to the rapid surface warming in the northern Arabian Sea and the adjacent northwest India and Pakistan, which emerge as the largest ocean and land surface trends in the South Asian domain. Though previous studies indicate an increase in spatial variability in these extreme rain events, we discover that they are nearly homogeneous across central India, from the west coast of Gujarat/Maharashtra to the east coast of Odisha (more than 500,000 km^2^ in area).

## Results

### Increase in extreme events

Though the increase in extreme rain events are spread over a large region across the subcontinent, we focus on the central Indian region (76°–86° E, 19°–26° N) where the mean monsoon rainfall is exhibiting a decline and where about 60% of the agriculture is rain-fed (Fig. 1a). Figure 1c shows the time series of the mean summer monsoon rainfall during 1950–2015, indicating a 10% decline in the rainfall averaged over the central Indian region, with 20–30% decrease over some of the grid points (Fig. 1a). Meanwhile, the frequency of extreme rainfall events (daily rainfall ≥ 150 mm, see “Methods”) over central India has increased by about 75% during the period (significant at 95% confidence level, Fig. 1b, e). This means that the extreme events are on the rise at a rate of about 13 events per decade (more than one per year). Further, we note that it is not only the frequency of extreme rain events which are increasing, but the extremes themselves are intensifying over time—indicated by an increase in the 99.5th percentile values (Supplementary Fig. 1d). The increase in extreme rainfall while the mean rainfall is decreasing also implies that the dry spells may be increasing in this region^2^. In fact, the 75th percentile values of rainfall (moderate rain events) show a decline over time, suggesting that the rainfall distribution is becoming wider and flatter. Despite several studies^5, 7, 24–27^ indicating increased spatial variability of these events over the subcontinent, the analysis here suggests a homogeneous spatial extent of the increasing trend in extreme events across a large area over central India, from the west coast of Gujarat/Maharashtra to the east coast of Odisha (Fig. 1b, 70–90 E, 18–26 N, > 500,000 km^2^ in area).

Though several earlier studies suggest that the rise in extreme rainfall events over central India is due to an increase in the moisture content^14–16^, our analysis (Fig. 1d) indicates that the moisture levels in the atmospheric column (1000–200 hPa) over central India have in fact reduced during 1950–2015—though with an increase in the recent decade. In addition, a recent study using station data shows that the increase in specific humidity anomalies in the recent decade is lowest during the summer monsoon^45^. Meanwhile, the number of extreme rainfall events and the surface temperatures over central India show a negative correlation (Supplementary Fig. 2a, b). This is intriguing because across most of the tropics and the monsoon regions in general, the local thermodynamics plays a major role in enhancing the extreme rainfall events^46^, regulated by the Clausius–Clapeyron relationship^47, 48^. However, it is interesting that the local changes of precipitation over the central Indian subcontinent are largely dominated by dynamic responses of the atmosphere rather than thermodynamic constraints^29^. This is evident from Supplementary Fig. 2a, b, which shows that the trend in extreme events shows a negative correlation for the local temperatures over the central Indian region, while it is statistically correlated with warming temperatures in other areas, especially over the Indian Ocean. Similarly, the daily maximum temperatures also show a negative relationship with the extreme rain events over central India^49^. Apart from these factors, the number of days during which depressions or cyclonic storms enter the central Indian region shows a decline, in conformity with earlier studies^38, 39^ (Fig. 1d). This indicates that the changes in frequency of depressions may not have a positive contribution in increasing the number of extreme rainfall events over the subcontinent.

Most of the studies dealing with the driving mechanisms of extreme rainfall events over central India have analyzed these events in the context of the changes in largescale conditions and correlation analysis on seasonal or annual time scales (Supplementary Fig. 2a, b). While instructive in terms of rudimentary information on the association between these events and largescale climatic conditions, they do not fully explore causality. For example, the monotonous rise in equatorial Indian Ocean sea surface temperatures (SST)^22, 50–52^ appears to be well correlated with the changes in extreme rainfall events^14, 15^ (Supplementary Fig. 2a). Nevertheless, this could be due to the fact that both the variables exhibit unidirectional trends. Goswami et al. and others^14, 15, 20^ postulate that the warming in the central Indian Ocean increases the water vapor in the atmosphere, which ultimately provides more moisture to the central Indian region. However, the correlation between extreme rainfall events and humidity levels over the central Indian Ocean appears weak and insignificant (Supplementary Fig. 2c). This could mean that though the rapid SST warming has indeed moistened the local atmosphere over the ocean, it has not resulted in increased rainfall over central India—possibly due to a weakened monsoon circulation^12^. It also points to the complex nature of the dynamics involved, requiring an analysis on daily timescales in order to disentangle the role of the Indian Ocean on the increased frequency of the extreme events.

### Widespread extreme events

In order to elucidate the causality of the extreme rainfall events and to trace the source of moisture feeding these episodes, we investigate the daily evolution of the hydroclimatic conditions leading to these events. For this analysis, we define widespread extreme rainfall events as those days with extreme events occurring simultaneously on ten or more grid points—such that they cover a sufficiently large area and can cause largescale floods^34^. Also, by examining extreme daily precipitation events that cover several grid points or observational stations over central India, we eliminate sporadic extreme events which exhibit large spatial heterogeneity due to localized factors and do not pass the field significance test^5, 24^.

The frequency of widespread extreme events exhibits inter-decadal fluctuations, similar to the variability observed by earlier studies^15^ (Fig. 1f). Despite the inter-decadal fluctuations, the trend is consistent with a threefold increase in the number of widespread extreme events over central India during 1950–2012. These trends in extremes are found to be homogenous across the central belt of India (field significant at 95% confidence level, see “Methods”). Identifying homogeneous regions offers clearer pathways for exploring predictions and projections of these events and their trends since predicting individual events is a bigger task. Henceforth, we investigate the uniform nature of these events which display larger scale variability. Studying the precursory conditions of extreme events requires high resolution data both in temporal and spatial scales and hence we resort to assessing the daily data of hydroclimatic parameters during the satellite era, from 1982 to 2015.

### Increasing variability of monsoon westerlies

**Fig. 2:**
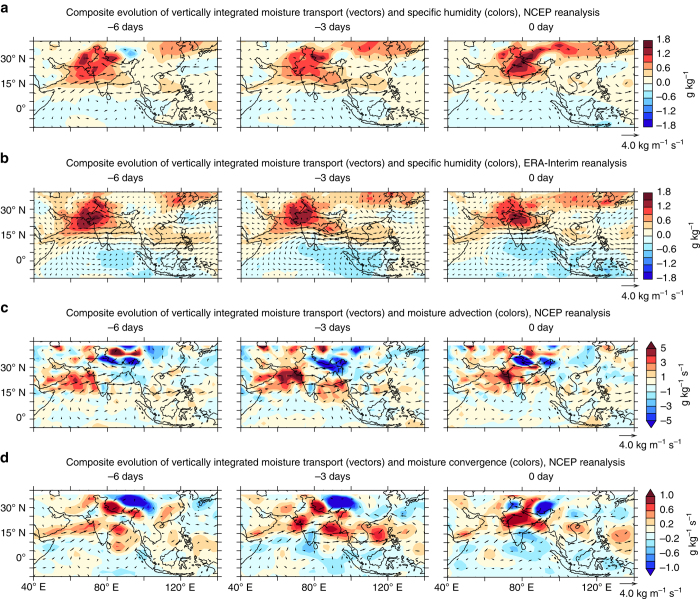
Evolution of moisture transport leading to widespread extreme precipitation events. Composite evolution of moisture transport (kg m^−1^ s^−1^, vectors) and **a**, **b** specific humidity (g kg^−1^, colors), **c** moisture advection (10^−6^ g kg^−1^ s^−1^, colors) and **d** moisture convergence (10^−5^ g kg^−1^ s^−1^, colors), leading to widespread extreme precipitation events (more than ten grid cells with daily rainfall ≥ 150 mm) over central India (on 0-day). Daily data for the period 1982–2015 is used for the composite analysis, based on NCEP (**a**, **c**, **d**) and ERA-Interim reanalysis (**b**). The analysis is for vertically integrated (1000–200 hPa) values for summer (June–September)

Since the key unresolved factor in the case of increasing extreme rain events is the source of moisture and the mechanism by which the moisture is transported to the central Indian region, we examine daily composite maps of the moisture levels and transport preceding the widespread extreme rainfall events. The widespread extreme events are generally spread over a period of 2–3 days, and the composites are prepared with respect to the peak day. Figure 2a indicates positive specific humidity anomalies (averaged over 1000–500 hPa) developing over the northern Arabian Sea region prior to an extreme event. The composites show nearly stationary but accumulating levels of humidity anomalies over the region up to 6 days prior to the event (Fig. 2a). Thereafter the strengthening westerlies transport the moisture from the Arabian Sea towards the central Indian region, engendering intensified precipitation over the subcontinent. The evolution of these events analyzed using a different reanalysis data set (ERA-Interim, Fig. 2b) portray almost identical composites, indicating the robustness of the current results. Further, we prepared the dynamical breakup of the source of moisture, to examine the relative roles of horizontal moisture advection and convergence separately^53, 54^. The results confirm enhanced moisture advection from northwestern Arabian Sea along the path of the westerlies (Fig. 2c), which converge over central India (Fig. 2d), resulting in extreme rain events.

**Fig. 3:**
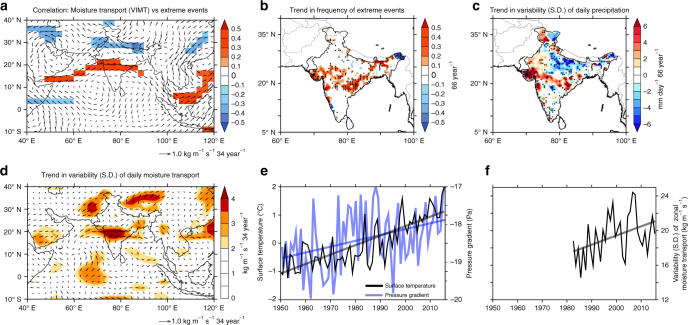
Variability in moisture transport vs widespread extreme precipitation events. **a** Correlation between the number of extreme precipitation events over central Indian subcontinent (75°–85° E, 19°–26° N, inset boxes in Fig. 1) and vertically integrated (1000–200 hPa) moisture transport (vectors), for the years 1950–2015. The color shades indicate the correlation with the zonal component of the moisture transport at each grid point. **b** Trend in the frequency of extreme precipitation events (66 year^−1^), for the years 1950–2015. Trend in the standard deviations (S.D.) of **c** daily precipitation (mm day^−1^ 66 year^−1^) and **d** daily vertically integrated moisture transport (kg m^−1^ s^−1^ 34 year^−1^, vectors) during 1982–2015. The color shades indicate the trend in the S.D. of zonal component of the moisture transport at each grid point (kg m^−1^ s^−1^ 34 year^−1^). **e** Time series of the surface temperatures averaged over north of Arabian Sea (50°–65° E, 15°–35° N, °C, black line), and cross-equatorial pressure gradient (Pa, blue line)^60^, which is estimated as the difference of mean sea level pressure (MSLP) between (20°–35° N, 40–80° E) and (35°–10° S, 40°–80° E). **f** Time series of standard deviations of daily zonal moisture transport (moist westerlies, kg m^−1^ s^−1^). Color shades in **a**, **d** and stippling in **b**, **c** indicates correlation/trends significant at 95% confidence level. The trend lines shown in the figures are significant at 95% confidence level. Analysis of daily data is restricted to the satellite era. The precipitation data is based on IMD observations, the moisture transport and MSLP is obtained from NCEP reanalysis, and the surface temperatures from CRU/HadISST. See the “Methods” section for more information regarding the data

A correlation analysis of the moisture transport anomalies with the frequency of extreme precipitation events (Fig. 3a) yield maximum significant correlation coefficients for the westerly moisture transport from the Arabian Sea. The strong positive correlation with the moisture transport along with a consistent decrease in the precipitation and humidity levels (Fig. 1c, d) suggests a rise in the daily variability (variability on daily timescales) of the moist westerlies. In fact, a trend analysis of daily precipitation variability (Fig. 3c) indicates increasing fluctuations in precipitation, closely adhering to the pathway of these intense surges of moisture transport covering entire central India (Fig. 3d). Indeed, the spatial distribution of the trends in the frequency of extreme events (Fig. 3b) show coherency with the daily variability of the moist westerlies, confirming its role in intensifying the precipitation events.

### Role of low-pressure systems

Earlier studies have indicated that low-pressure systems which develop in the Bay of Bengal and move northwestward into central India are a key source of moisture for the extreme events^18, 31, 32, 34–36^. Subsequent to an intensification of the moist westerlies over central India, a short-lived cyclonic system also develops over the Bay of Bengal, 3 days prior to the extreme event (Fig. 2, at 3 days’ lead). This system does not sustain through the next triad (period of 3 days), and vanishes without deepening into a depression or a cyclonic storm. Some studies^55^ suggest that an increase in similar synoptic disturbances termed as lows (wind speed < 8.5 m s^−1^), which correlate with the rising frequency of extreme rain events, outweigh the declining trend in the number of depressions. However, the current analysis (Fig. 2) indicate that these weak low-pressure systems do not transport sufficient moisture into the central Indian region probably because they do not deepen to strong moisture carrying cyclonic storms in the Bay of Bengal. In fact, the episodic surges in moisture transport from the Arabian Sea might be playing an increasing role in fostering these weak low-pressure systems in the Bay of Bengal (Fig. 2). This has been pointed out by earlier studies^31^, which suggest that the intensification of the Bay of Bengal low-pressure systems is marked by increased influx of moisture from the Arabian Sea, along with the strengthening of the monsoon westerlies. Bay of Bengal is a region where the precipitation exceeds evaporation during summer, and this is maintained by a substantial moisture influx from the Arabian Sea^39^. This moisture influx towards the Bay of Bengal has reduced in the recent decades, which may have adversely affected the genesis and intensification of monsoon depressions^39^. These results indicate that the atmospheric humidity levels evolving over the Arabian Sea and transported by the enhanced westerlies have a major role in nurturing intensified rains over central India. Nevertheless, it is to be noted that the low-pressure systems can be instrumental in inducing a strong moisture convergence which is key for the occurrence of the extreme events (Fig. 2d).

### Source of moisture for the widespread extreme rain events

**Fig. 4:**
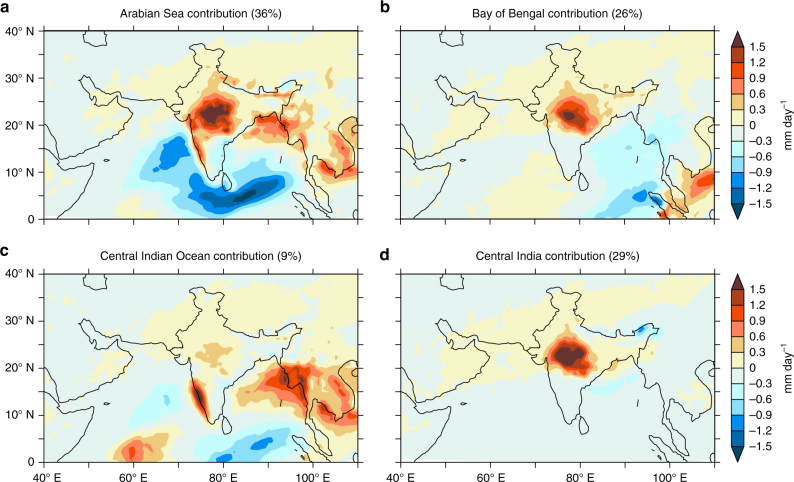
Moisture contribution from ocean and terrestrial sources. Contribution of **a** Arabian Sea (50°–75° E, 5° S–30° N), **b** Bay of Bengal (80°–100° E, 5° S–30° N), **c** central Indian Ocean (50°–100° E, 25°–5° S) and **d** central Indian subcontinent (73°–83° E, 16°–28° N) in supplying moisture for the extreme precipitation events over central Indian subcontinent. The moisture contribution is estimated using the Dynamic Recycling Model. The colors indicate individual source’s contribution to the precipitation anomaly in mm day^−1^. The negative precipitation anomalies (mm day^−1^) south of the subcontinent indicate deficient rainfall because of the northward shift in rainfall bands during extreme rainfall events over central India. The anomaly is calculated as deviation from the seasonal mean precipitation. The percentages in the subheadings show the fractional contributions of moisture to the extreme precipitation over the selected region in central India from different sources

To ascertain the source of moisture during extreme rainfall events, we use a dynamic recycling model (DRM)^56^ based on a Lagrangian trajectory approach, where the water vapor prior to precipitation over a region is traced backward in time and the contribution from each source is quantified (Fig. 4). The model tracks the moisture contributions from various sources, including those from lows, depressions, and moist westerlies. The results indicate that ∼36% of the total moisture for these extreme events comes from the Arabian Sea, while only 26% is traced to the Bay of Bengal and 9% to the central Indian Ocean. This means that the Arabian Sea supplies more moisture to the extreme rain events than the Bay of Bengal and the central Indian Ocean combined. A Mann–Kendall trend analysis indicates a positive trend (Supplementary Table 1, Theil–Sen’s slope = 0.06), in the moisture contribution from the Arabian Sea, significant at 95% confidence level. Meanwhile, the local recycling (i.e., moisture evaporated from central subcontinent and precipitates locally) contributes to about 29% of the moisture, slightly larger than the Bay of Bengal contribution. Earlier studies^57, 58^ have shown that land surface processes and local recycling is a significant factor for the summer monsoon over central India, contributing up to 25% of the moisture.

### Precursors for prediction of widespread extreme rain events

**Fig. 5:**
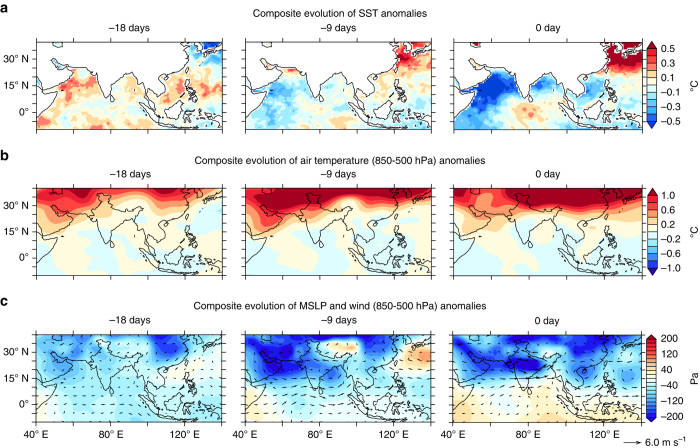
Evolution of temperature, pressure and winds leading to widespread extreme precipitation events. Composite evolution of **a** sea surface temperature (SST) and **b** air temperature (850–500 hPa) anomalies (colors, °C), **c** mean sea level pressure (MSLP, colors, Pa) and wind (vectors, m s^−1^), leading to widespread extreme precipitation events over central India (on 0-day). Daily data from OISST and NCEP reanalysis, for the period 1982–2015 is used for the composite analysis

**Fig. 6:**
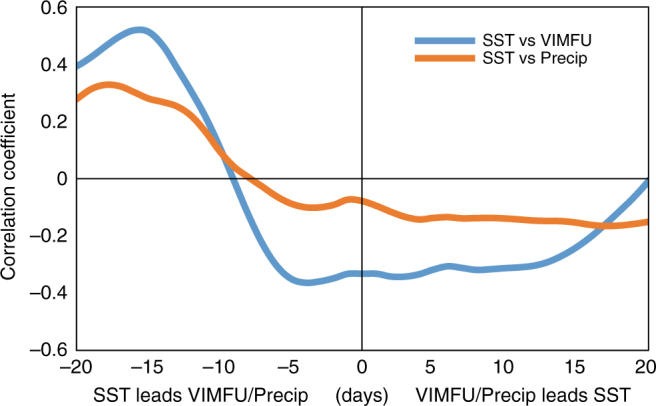
Lead–lag analysis of sea surface temperature vs moisture transport and precipitation. Lead-lag correlation between sea surface temperature (SST) and the zonal component of the vertically integrated moisture flux (VIMFU, moist westerlies) and precipitation. SST is averaged over the northern Arabian Sea (55°–70° E, 15°–23° N), and VIMFU (70°–80° E, 18°–22° N) and the precipitation (76°–86° E, 19°–26° N) over central India where it shows the maximum correlation with the SST. The SST anomalies show maximum correlation with the moist westerlies and rain events over central India at 2–3 weeks lead

The extreme rainfall events are preceded by warm SST anomalies over the northern Arabian Sea, at 2–3 weeks’ lead (Fig. 5a). The mean summer SSTs in the northern Arabian Sea are about 28°–29 °C which are highly conducive for active convection, and warm SST anomalies further enhance the humidity and convective activity in the tropospheric column^59^ (Fig. 2). Apart from the warm SST anomalies, the air temperature anomalies indicate a strengthening of the heat low over Pakistan and northwestern India (Fig. 5b). A combination of these warm land and ocean temperatures to the north of Arabian Sea (15° N) enhances the meridional thermal and pressure gradients over this location. Since the region is away from the equator, the Coriolis force is stronger and hence intensifies a westerly geostrophic flow orthogonal to the pressure gradient (Fig. 5c)^60^. The SST anomalies over the north Arabian Sea show maximum correlation with the moist westerlies and rain events over central India at 16 and 19 days’ lead, respectively (at 2–3 weeks), and provides a useful precursor to the widespread extreme rain events (Fig. 6). Further analysis is required to assess the predictability of these events. Meanwhile, it is evident from the composite analysis in Fig. 5, that local temperatures do not show an increase during or before an extreme event at the daily timescale, further suggesting that the extreme rain events over central India are dominated by dynamic responses of the atmosphere rather than thermodynamic constraints.

### Widespread extreme rain events and historical floods

We analyzed some of the historical floods (International Disaster Data Base) which resulted in the largest loss in terms of human lives and property—and which coincided with the occurrence of some of the strongest widespread extreme events. These individual events (Supplementary Fig. 3) show identical evolution as in the composites (Figs. 2 and 5). For example, the central Indian floods in 1989 and 2000, Mumbai floods in 2005, South Asian floods in 2007, etc. were a result of intense convective precipitation spread over a period of 3 days, tracing their moisture source to the northwesterly flow from the Arabian Sea (Supplementary Fig. 3). In fact, a handful of studies on the Mumbai floods in 2005 indicate that the extreme rain event was linked to an active moisture flow from the Arabian Sea, consistent with our results^61^.

These events were not spatially isolated events but wide-spread, with some of these events spanning across the entire central subcontinent from the west coast of Gujarat and Maharashtra to the east coast of Odisha (68°–85 °E, 16°–26 °N). All of these events were preceded by strong positive SST anomalies at 2–3 weeks lead and in the range of 1–2 °C and above, over the north Arabian Sea (Supplementary Fig. 3). Numerical model experiments^62^ indicate that warmer SST anomalies of the order of 1 °–2 °C in the northern Arabian Sea cause surface pressure to drop by 2–3 hPa, leading to local acceleration of winds (1–5 m s^−1^), rise in evaporation (20–40%) and increased variability in mesoscale circulations. Further, we performed the moisture tracking analysis using the DRM, for the individual events (Supplementary Fig. 4). The individual cases show that the Arabian Sea contribution is much larger than the entire contribution from the Bay of Bengal—even when these events coincided with the lows and depressions moving into the central Indian region. Also, it appears that a large share of the moisture content over the Bay of Bengal during these events is supplied remotely from the Arabian Sea, rather than local sources.

### Potential role of land–ocean warming

Summertime SST trends for the period show rapid warming of up to 1.5 °C in the northern Arabian Sea (Supplementary Fig. 5a, b), indicating its potential role on the increased daily atmospheric variability in the region (Fig. 3c, d, f). To the north of this region, the land surface temperatures in the Indo-Pak thermal low region also exhibits strong warming trends of up to 3 °C (Supplementary Fig. 5c, d). These two regions north of the Arabian Sea emerge as the largest surface temperature trends in the ocean and land over the South Asian domain (40°–120° E, 20° S–50° N). In fact, an earlier study^60^ indicates that rapid warming of surface temperatures north of Arabian Sea (20°–35° N) deepens the low-pressure area and subsequently strengthens the meridional pressure gradient over the region. This results in a strengthening of the low-level westerlies north of 15 °N over the Arabian Sea^60^, which is collocated with the observed changes in the westerlies in our study. Figure 3e, f shows the time series of surface temperature averaged over the north of Arabian Sea, cross-equatorial pressure gradient^60^, and the variability in the zonal moisture transport (moist westerlies) over central Indian subcontinent. The surface temperature anomalies show an increasing trend, and a significant correlation (at 95% confidence levels) with the increasing pressure gradient anomalies (*r* = 0.36) and the variability of the moist westerlies (*r* = 0.33).

## Discussion

**Fig. 7:**
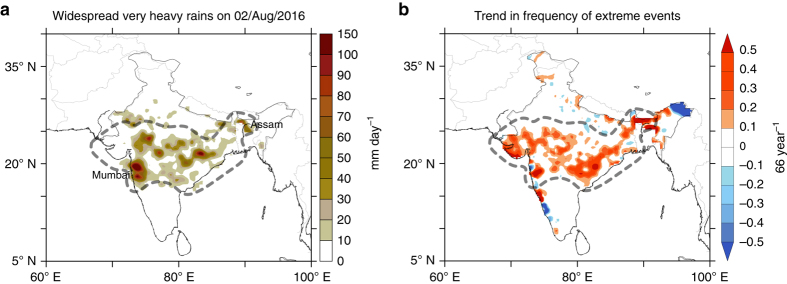
Precipitation distribution during the recent floods over central India. **a** Precipitation (mm day^−1^) on 2nd August 2016, which resulted in widespread floods across India. Precipitation values are based on the TRMM Multi-satellite Precipitation Analysis (TMPA, 3b42). **b** Observed trend in the number of extreme precipitation events during 1950–2015. Dashed line indicates the central Indian belt where the widespread extreme rain events are increasing

Recently, during the summer of 2016, heavy-to-extreme rainfall events occurred across the central Indian region resulting in largescale floods. The floods were widespread, and caused damage to life and property in the west coast including Mumbai, and submerged the Kaziranga National Park in the state of Assam in northeastern India^63^. A snapshot of the satellite rainfall data during these floods reveals a widespread heavy rainfall event spread over a period of 3 days (Fig. 7a). The regional extent of the widespread rainfall event shows a huge similarity with that of the increasing trends in extreme rain events (Fig. 7b).

CMIP5 projections do indicate further warming in the Arabian Sea^22^, which might increase the number of such extreme events in the future, putting lives and the agriculture sector at risk. Nevertheless, the spatial homogeneity of these widespread events, and their significant association with the positive SST anomalies in the Arabian Sea offers an opportunity in forecasting these events with a potential predictability of about 2–3 weeks in advance. Webster^64^ points out that the cost of extended 2-week forecasts for South Asia for hydrometeorological hazards including monsoon floods is relatively small, amounting to a maximum of $3 million per year. Considering that the economic losses due to these disasters exceed $3 billion per year (for the period 2006–2015, International Disaster Data Base), there is an urgent need to exploit the predictability of these widespread extremes and in improving their forecasts.

Most of the state-of-the-art models have cold SST biases in the Arabian Sea^65^, and the historical climate simulations using observed greenhouse gases forcing in these models do not reproduce the observed warming pattern in this basin^22^. These biases have a direct influence on the monsoon rainfall^65^ and its changes^66^, and hence it is necessary to reduce these biases in order to improve the model simulations of extreme rainfall events. Recently, an extended range prediction system has been setup under the National Monsoon Mission of the Government of India^67^, for providing meteorological forecasts more than 10 days in advance. The model utilizes daily SST values which are bias-corrected with respect to observed SSTs^67^, and has shown a reasonable skill in simulating some of the extreme rainfall events over South Asia^68^. If these extended range forecasts can be improved to predict the widespread extreme rainfall events, and are coupled to a hydrological model, then the high risk of extensive and prolonged flooding could be anticipated and actions taken to mitigate their impact^69^.

## Methods

### Rainfall and climate data analysis

The daily gridded rainfall data, at 0.25° horizontal resolution, for the northern summer during the period 1950–2015 is obtained from the India Meteorological Department (IMD)^70^. This includes rainfall records from 6955 stations for the period 1901 to 2015, which is the highest number of stations used by any gridded data sets currently available. The data density varies over time from about 1500 in the first few years to about 4000 by the end of the century. For the current analysis we use the data for the period 1950–2015 during which the data density was relatively higher, with more than 3100 stations per day^70^. It may be noted that a changing rain-gauge network may induce artificial trends in the extreme rainfall time series, compared to a fixed network of rain-gauges. However, our analysis is comparable with earlier studies which use fixed rain-gauge data^15^, and also with the trends in extreme events in the APHRODITE rainfall data set.

Rainfall events which exceed a threshold of 150 mm day^−1^ (in a 0.25 × 0.25° grid point) are counted as extreme rainfall events^14, 15^. Although a fixed threshold is not appropriate for defining extreme events over regions with large spatial variability in the mean climate, it can be useful in defining extreme rain events over central India, where the mean climate and daily variability is reasonably homogeneous^14^ (Supplementary Fig. 1a). Besides, according to WMO guidelines^71^, fixed absolute values represent the disaster-causing potential (floods, in the current context) better than percentile values. A comparison of extreme events based on fixed (150 mm day^−1^) and percentile (99.5%) thresholds yields similar variability and trends with a correlation coefficient of 0.97 (Supplementary Fig. 1b). The widespread extreme rain events in the study are defined as those days when the extreme events (daily rainfall ≥ 150 mm) simultaneously cover a sufficiently large area (ten grid cells or more) such that they can cause largescale floods^34^. In this case, the entire event in a day spread over the contiguous grid cells (box in Fig. 1b) is counted as a single widespread extreme event.

In order to examine the evolution of SST leading to the widespread extreme rainfall events, we use the NOAA 0.25° daily optimum interpolation sea surface temperature (OISST) for the period 1982–2015. The evolution of hydroclimatic conditions are investigated using daily values of specific humidity, air temperature and winds in the tropospheric column, geopotential height (500 hPa) and mean sea level pressure from NCEP reanalysis, during the same period. The depression tracks and frequency data is based on the “Cyclone eAtlas—IMD” published by the IMD. A recent study using an objective application of cyclone tracking on reanalysis suggest possible errors in the IMD depression data over the ocean, attributed to poor observations or the algorithm used for objective tracking over the ocean^55, 72^. Nevertheless, our analysis is based on depressions reaching (or occurring over) the central Indian subcontinent, where the detailed local information provided by the IMD observations provides a more accurate account of depressions in this region.

### Moisture transport and flux convergence

The vertically integrated moisture transport (**Q**) gives a measure of forced lifting and moisture supply, and is calculated by summation of the horizontal moisture transport over the troposphere (1000–200 hPa), i.e.,

$${\bf{Q}} = \frac{1}{g}\mathop {\int }\nolimits_{{P_{200}}}^{{P_{1000}}} q{\bf{V}}{\rm{d}}P,$$

where *q* is the specific humidity, **V** is the wind vector, *P* is the pressure, and *g* is the acceleration due to gravity. The significance of the trends and correlations is examined using standard two-tailed Student’s *t*-tests. In order to avoid any potential time-variable errors in the estimates of humidity and moisture transport, we perform additional analysis using specific humidity and wind fields based on the ERA-interim reanalysis for the period 1982–2015.

In order to examine the relative roles of horizontal moisture advection and convergence separately, we prepared the dynamical breakup of the horizontal moisture flux convergence (MFC)^53, 54^. The horizontal MFC can be written as:

$$\begin{array}{rcl}{\rm{MFC}} = { - \nabla .\left( {q{{\bf{V}}_h}} \right) = - }_{}{{{\bf{V}}_h}.\nabla q - q\nabla .{{\bf{V}}_h}}_{}\\ \\ {\rm{MFC}} =- \underbrace{ u\frac{{{\rm{d}}q}}{{{\rm{d}}x}} - v\frac{{{\rm{d}}q}}{{{\rm{d}}y}}}_{\text{advection term}} - \underbrace{q\left( {\frac{{{\rm{d}}u}}{{{\rm{d}}x}} + \frac{{{\rm{d}}v}}{{{\rm{d}}y}}} \right)}_{\text{convergence term}}\\ \end{array}$$

The advection term represents the horizontal advection of specific humidity. The convergence term denotes the product of specific humidity and horizontal mass convergence.

### Field significance tests

To evaluate the homogeneity of the trends in widespread extreme events over the central belt of India, we conducted a field significance test following Krishnamurthy et al.^24^. Though Goswami et al.^14^ reported increasing trends over central India, this was contested by later studies^5, 24, 25^ indicating spatial heterogeneity in the trends of extreme events over central Indian region. However, this heterogeneity appears because they utilized a rigid box where opposing trends due to local thermodynamic and remote dynamic factors are involved. Here we select an irregular region where the increase in extreme events is positive and the correlation with the moist westerlies is significant (Fig. 3a), and conduct the field significance test on the trends in extreme rainfall events over this region. Ideally, this would be the region which would witness an intensification of the extreme events due to changes in the westerlies. The field significance test is considered to be passed if majority of the grids in a region have a statistically significant trend at 95% confidence level and their collective trend is not by chance due to the spatial structure of the precipitation field. We perform bootstrapping to generate 1000 random samples of gridded annual/seasonal frequency data set and perform field significance test, where individual trend analyses of the grids are carried out with Mann–Kendall method to consider both linear and non-linear trends. We find that the trend of frequency of extreme events over the region has statistically significant increasing trend at 95% confidence level. Earlier studies have suggested increasing heterogeneity within the rigid central Indian box^5, 24^, while we find that there is homogeneity along the central belt where the moist westerlies show increased variability.

### Dynamic recycling model

The DRM uses the Lagrangian solution for the equation of conservation of the total column water vapor and quantifies the relative contributions from different sources to the atmospheric moisture over a given sink region^56, 57, 73^. Daily averages of evaporation, total column water and precipitation from the ERA-interim reanalysis data set are used as input to the DRM. In the current study, the source regions are identified as Arabian Sea (50°–75° E, 5°–30° N), Bay of Bengal (80°–100° E, 5°–30° N), equatorial Indian Ocean (50°–100° E, 10° S–5° N), and central Indian subcontinent (73°–83° E, 16°–28° N). The potential error in tracking by this method is about 5–10% in those cases where the DRM estimates are influenced by the effect of vertical wind shear^56^. This is because the transport of moisture from local and remote sources can be in different directions when there is substantial vertical wind shear. Nonetheless, the transport of moisture to the central Indian subcontinent as estimated by the DRM is in agreement with the analysis of the hyrdroclimatic conditions leading to the extreme rainfall events. It is also to be noted that the fractions of moisture contributions from different sources are sensitive to the selection of the reanalysis, based on the physical parameterizations utilized in each product. Even though there is uncertainty in the results of DRM across reanalysis products, the relative contribution of one source with respect to another remains unchanged^57^. See Supplementary methods on DRM for more details.

### Code availability

The DRM is developed based on Martinez and Dominguez^56^ and may be obtained from the original authors through personal requests. The Matlab code used to post process the output, extract the moisture fraction and calculate the contribution during extreme precipitation days have been archived by the authors and are available on request from the author S.G., subimal@civil.iitb.ac.in.

### Data availability

The rainfall data used in the study for the period 1950–2015 is obtained from the IMD (http://www.imd.gov.in/pages/advertisements_view.php?ff=20170320_advt_34). The depression tracks and frequency data is based on the “Cyclone eAtlas—IMD” published by IMD (http://www.rmcchennaieatlas.tn.nic.in). The daily SST for the period 1982–2015 is the OISST, available from the National Oceanic and Atmospheric Administration (NOAA) (https://www.ncdc.noaa.gov/oisst). The daily values of specific humidity, air temperature, winds, geopotential height and mean sea level pressure are available for download from NOAA NCEP website (https://www.esrl.noaa.gov/psd/data/gridded/data.ncep.reanalysis.html).

## Electronic supplementary material

Supplementary Information
