# Supplementary material for: A threefold rise in widespread extreme rain events over central India
Source: Nat Commun. 2017 Oct 3;8:708. doi: 10.1038/s41467-017-00744-9 (PMC5626780; doi:10.1038/s41467-017-00744-9)
Supplement: Supplementary file 1 — Supplementary Information [file 41467_2017_744_MOESM1_ESM.pdf]

Description of Supplementary Files

File Name: Supplementary Information
Description: Supplementary Figures, Supplementary Table, Supplementary Methods and Supplementary References

Supplementary Methods

We have used the Dynamic Recycling Model (Martinez and Dominguez, 2014), which is a Lagrangian trajectory based approach to quantify the impact of atmospheric transport of water vapor from different evaporative sources on precipitation on the Indian summer monsoon rainfall. A detailed methodology of moisture tracking and quantification can be found in Dominguez et al., (2006), Dominguez and Kumar (2008) and Martinez and Dominguez (2014). The same model has been applied to Indian sub-continent (Pathak et al., 2014, 2016).

For example, the fraction of precipitation generated from two adjoining regions *A* and *B* altogether, can be calculated by following the trajectory of the water vapor backward in time from its current location $(x(t),y(t))$in region *A*, to the location at the border of region $A(x(\tau_1),y(\tau_1))$, and from $(x(\tau_1),y(\tau_1))$ to the location in region B $(x(\tau_2),y(\tau_2))$. The evaporation over these regions (*A* and *B*) supply moisture to the air column along the trajectory, which is used in the precipitation over sink region. Therefore the fraction of atmospheric water present in the trajectory between the points $(x(t),y(t))$ to the $(x(\tau_2),y(\tau_2))$, resulting from evaporation in *A* and *B* is given by:

$$R(\chi, \xi, t) = 1 - \exp\left[-\int_{\tau_2}^{t} \frac{\epsilon\,(\mathrm{x}, y, \tau)}{\omega(\mathrm{x}, y, \tau)} \partial\tau'\right] = R_A(\chi, \xi, t) + \alpha_A(\chi, \xi, t)\, R_B(\chi, \xi, t) \quad (1)$$

where,

$$R_A(\chi, \xi, t) = 1 - \exp\left[-\int_{\tau_1}^{t} \frac{\epsilon\,(\mathrm{x}, y, \tau)}{\omega(\mathrm{x}, y, \tau)} \partial\tau'\right], \quad (2)$$

$$R_B(\chi, \xi, t) = 1 - \exp\left[-\int_{\tau_2}^{\tau_1} \frac{\epsilon\,(\mathrm{x}, y, \tau)}{\omega(\mathrm{x}, y, \tau)} \partial\tau'\right], \quad (3)$$

$$\alpha_A(\chi, \xi, t) = 1 - R_A(\chi, \xi, t) \quad (4)$$

Where, $R_A$and R_B, represents the fraction of evaporated moisture collected from two regions (*A* and *B*) along the water vapor trajectory between the points $(x(t),y(t))$ to the $(x(\tau_2),y(\tau_2))$ whereas ε/ω represents the ratio of evaporation to the precipitable water along the respective trajectory. In addition to that α_A represents the fraction of evaporated moisture from region *B* along the trajectory, which is not lost (via precipitation) in the intermediate part of trajectory in region *A*. Similarly, the net moisture contributions from different sources along the trajectory of the water vapor, can be quantified by calculating the moisture fraction corresponding to each source region.

For a domain *D* which consists of *N* different evaporative sources, the fraction of moisture collected from *N* different source regions SR_1, SR_2, SR_3,......, SR_N, is represented by R_1, R_2, R_3,........., R_N, respectively. Therefore, the total contribution at sink (χ, ξ, t), from all the segments (S_i: S_i = *1, 2*......) that are within the source region 'SR_i', and along the trajectory of the water vapor, are grouped as:

$$a_{SR}(\chi,\xi,t)=\sum_{S_i\in SR_i}\left(\prod_{j=1}^{S_i-1}\alpha_j(\chi,\xi,t)\right)*R_{S_i}(\chi,\xi,t) \tag{5}$$

The moisture fraction computed with Eq. (5), is used to quantify the fraction of precipitation generated over the sink (χ,ξ,t) from any specific source region. The precipitation generated over a sink (χ,ξ,t) that results from evaporation over the source region SR_i for any day t is calculated as :

$$PR_{SR_i}(\chi,\xi,t)=a_{SR_i}(\chi,\xi,t)*P(\chi,\xi,t) \tag{6}$$

Here, $P(\chi,\xi,t)$, represents the total precipitation over the point (χ,ξ) during day *t*.

The contribution to the daily precipitation computed with Eq. (6) is then used to study the dominant sources of moisture for the extreme event precipitation. Figure 4 shows the anomaly of the moisture contributions from different dominant moisture sources to the extreme precipitation over central India. The figure presents the composites of moisture contributions computed over the extreme days. The percentages in the subheadings show the fractional contributions of moisture to the extreme precipitation over the selected region in Central India from different sources. Here we show the percentage contribution from four moisture sources, viz. Arabian Sea (50°-75°E, 5°S-30°N), Bay of Bengal (80°-100°E, 5°S-30°N), central Indian Ocean (50°-100°E, 25°-5°S) and central Indian subcontinent (73°-83°E, 16°-28°N).

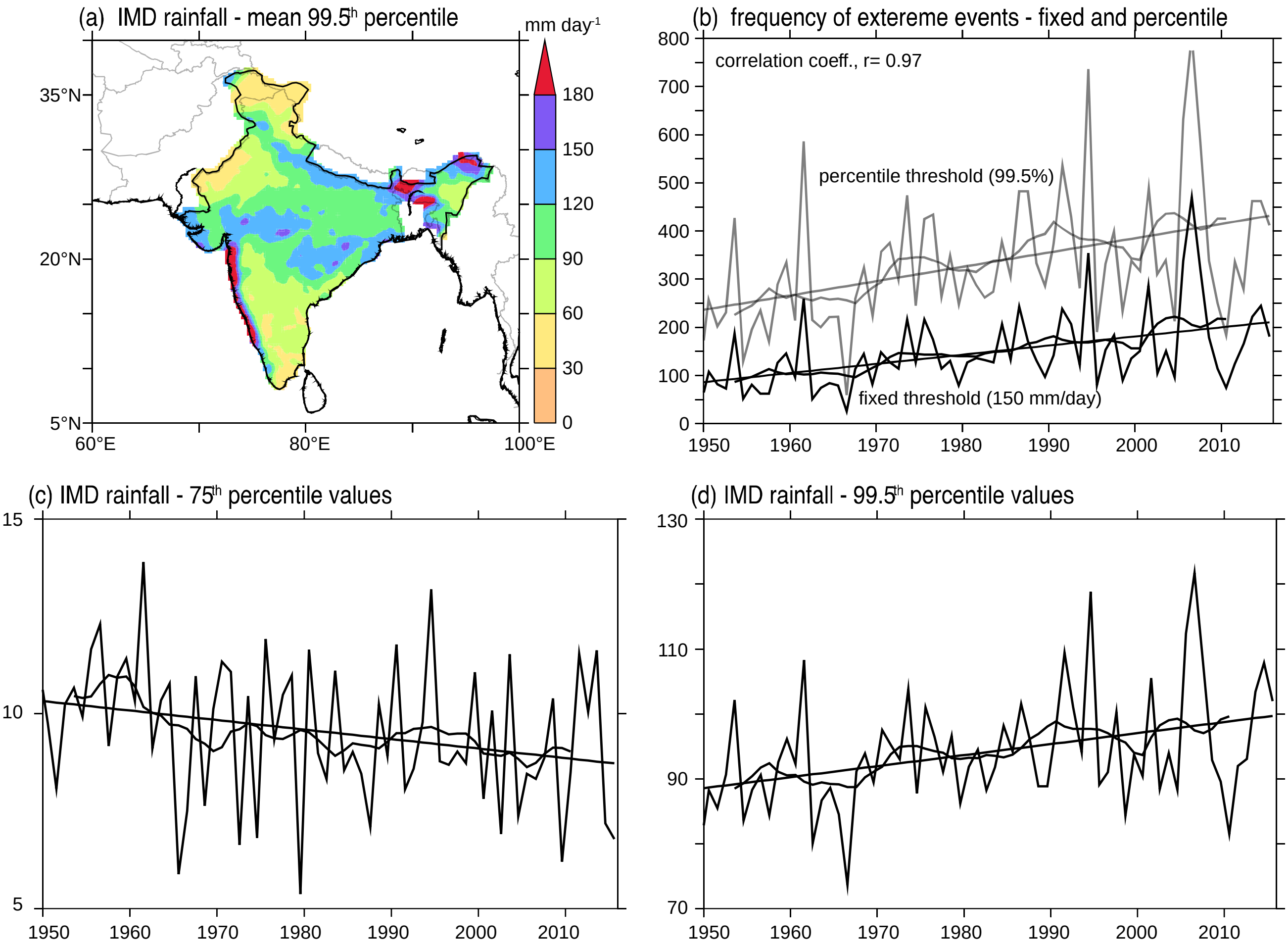

Supplementary Figure 1. (a) Spatial distribution of the 99.5th percentile values of rainfall (mm day^{-1}). (b) Time series of the frequency of extreme rain events over central Indian subcontinent (75°-85°E, 19°-26°N), where the extreme events are defined based on fixed (150 mm/day) and percentile (99.5%) thresholds. (c) 75th (moderate rainfall) and (d) 99.5th (extreme rainfall) percentile values of rainfall (mm day^{-1}). The data used is IMD daily gridded rainfall for summer, during 1950-2015.

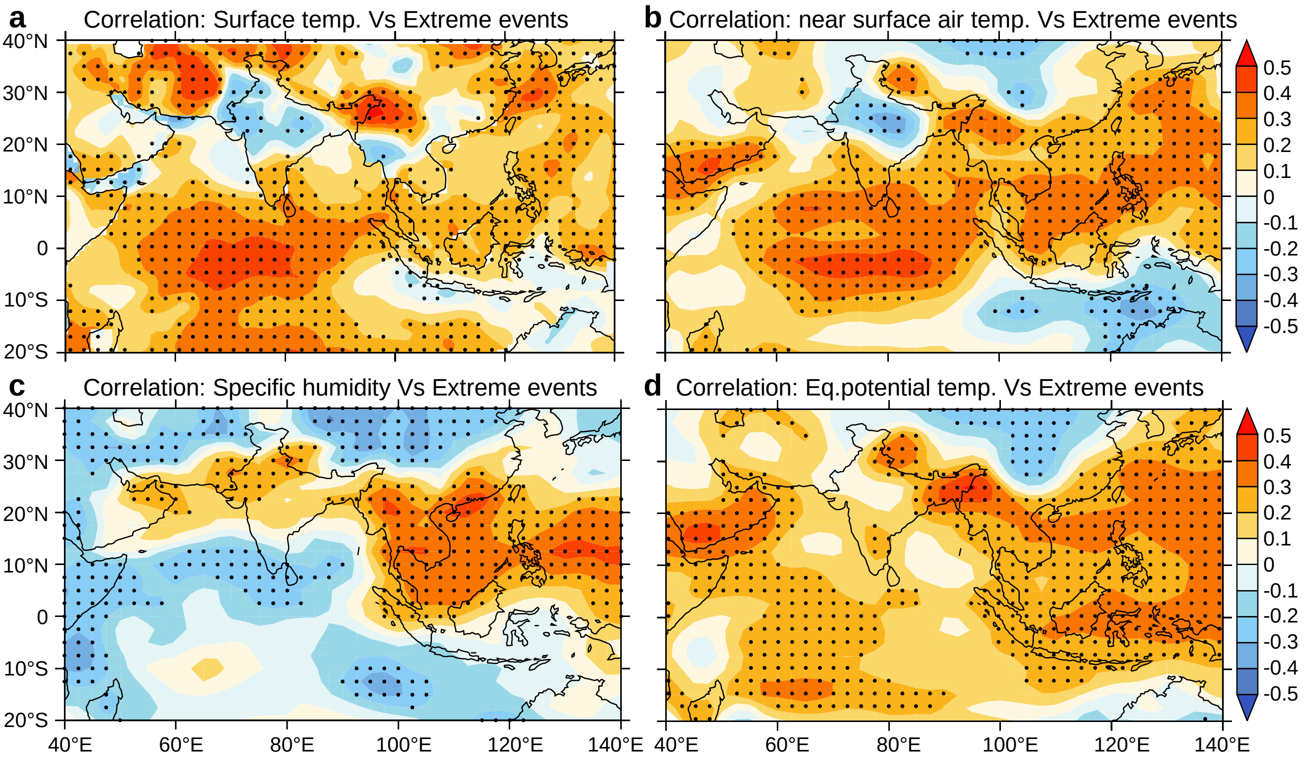

Supplementary Figure 2. Correlation between the number of extreme precipitation events over central Indian subcontinent (75°-85°E, 19°-26°N, inset boxes in Fig.1) and (**a**) surface temperature anomalies based on HadCRUT/HadISST observations and (**b**) near surface temperature, (**c**) specific humidity and (**d**) equivalent potential temperature anomalies based on NCEP reanalysis. Specific humidity and equivalent temperature anomalies are estimated for the atmospheric column over 1000-200 hPa. The correlation analysis is done between the frequency of extreme events and the seasonal mean (June-September) anomalies of temperature and humidity, for the years 1950-2015. Stippling indicates correlation coefficients significant at 95% confidence level.

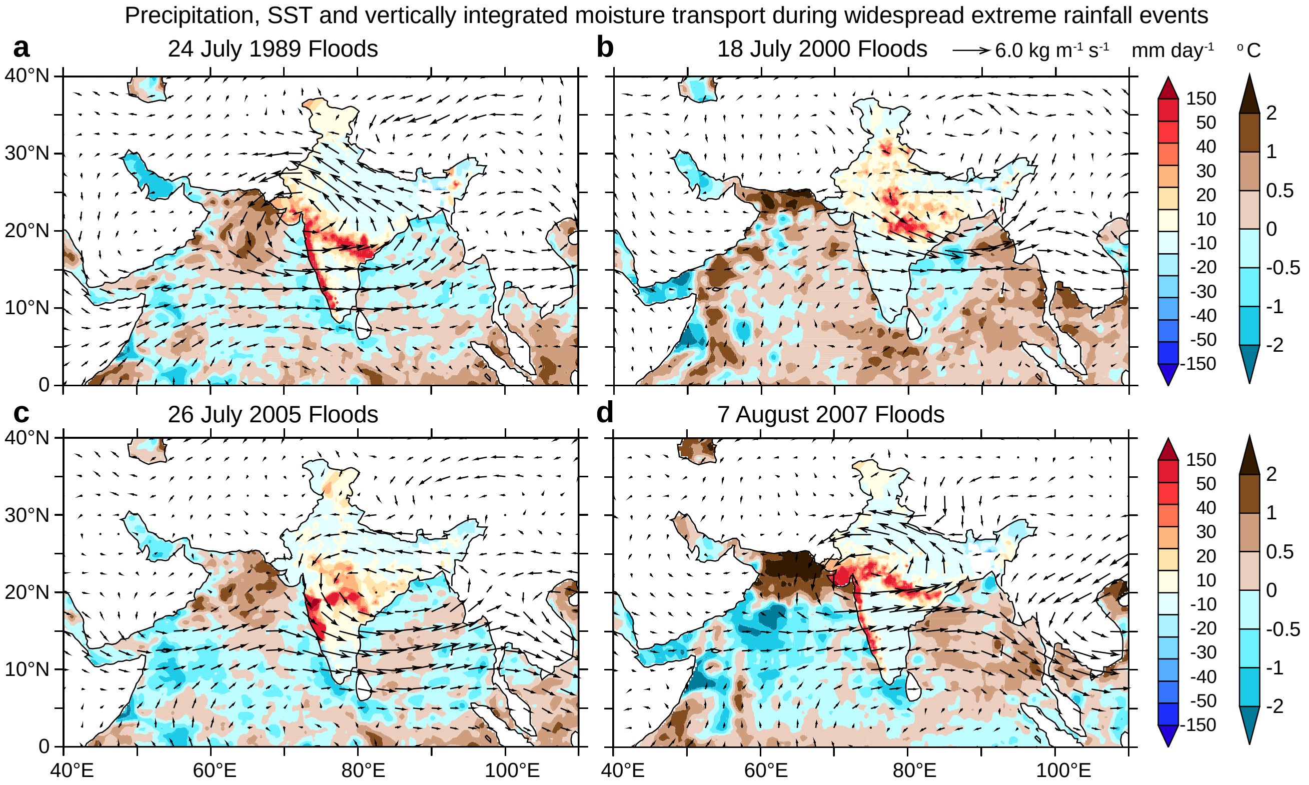

Supplementary Figure 3. Precipitation (mm day^{-1}, colors over land), SST (°C, colors over ocean) and vertically integrated moisture transport (1000–200 hPa, kg m^{-1} s^{-1}, vectors) anomalies during four widespread extreme precipitation events over the central Indian subcontinent—floods in the years (**a**) 1989 and (**b**) 2000, (**c**) Mumbai floods in 2005, and (**d**) South Asia floods in 2007. The SST anomalies shown are at 2-3 weeks (average) lead with respect to the peak day. The moist westerlies and the precipitation is a three-day average, centered on the peak day. The lead-lag relationship is estimated based on the analysis in Figure 6.

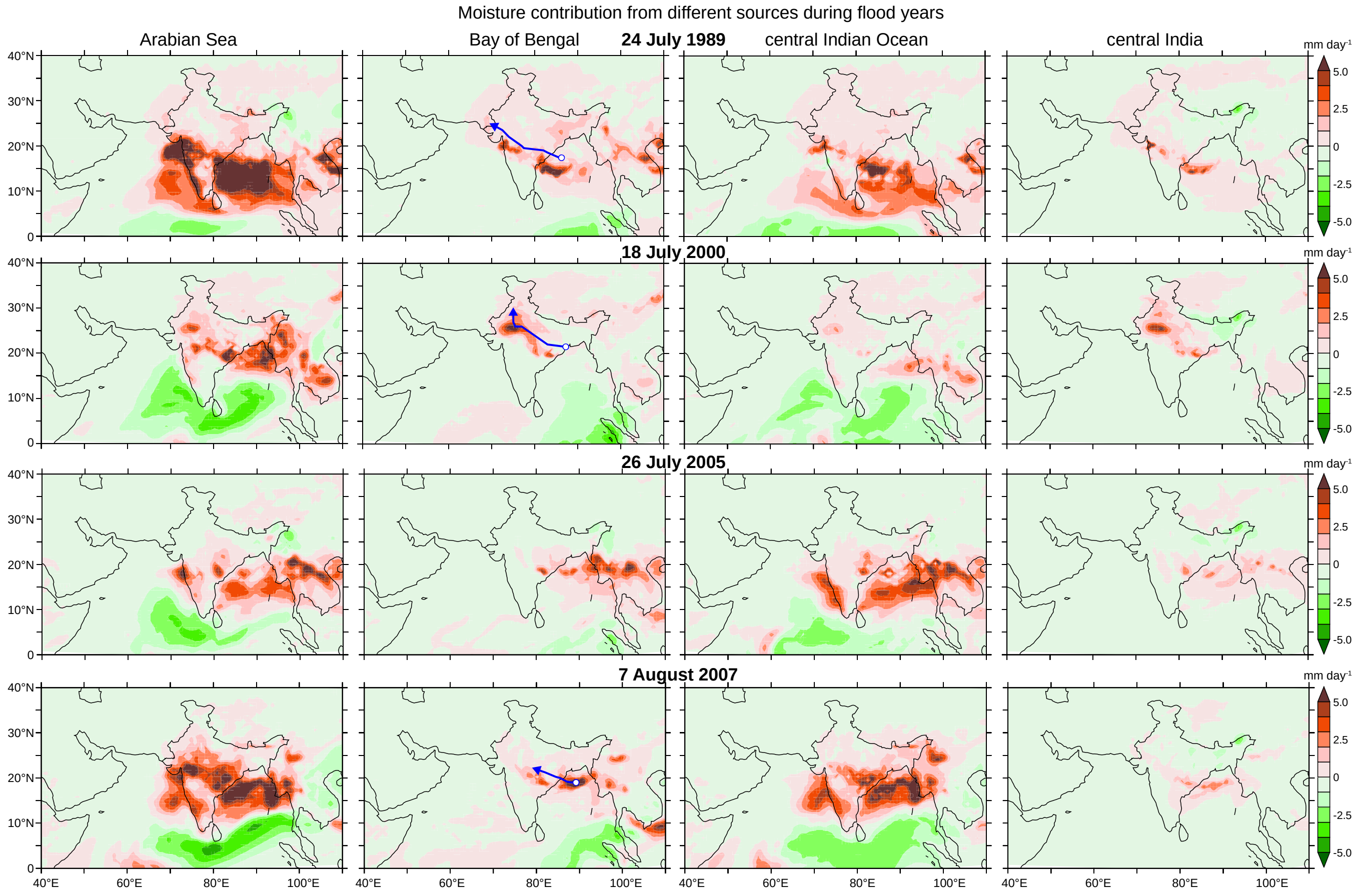

Supplementary Figure 4. Relative contributions of Arabian Sea (50°-75°E, 5°S-30°N), Bay of Bengal (80°-100°E, 5°S-30°N), central Indian Ocean (50°-100°E, 25°-5°S) and central India (73°-83°E, 16°-28°N) in supplying moisture for the flood events over central India. The moisture contribution is estimated using the Dynamic Recycling Model. The colors indicate individual source's contribution to the precipitation anomaly in mm day^{-1}. The blue line indicates the track of low pressure systems (lows or depressions) originating in the Bay of Bengal and reaching central India, during the flood events.

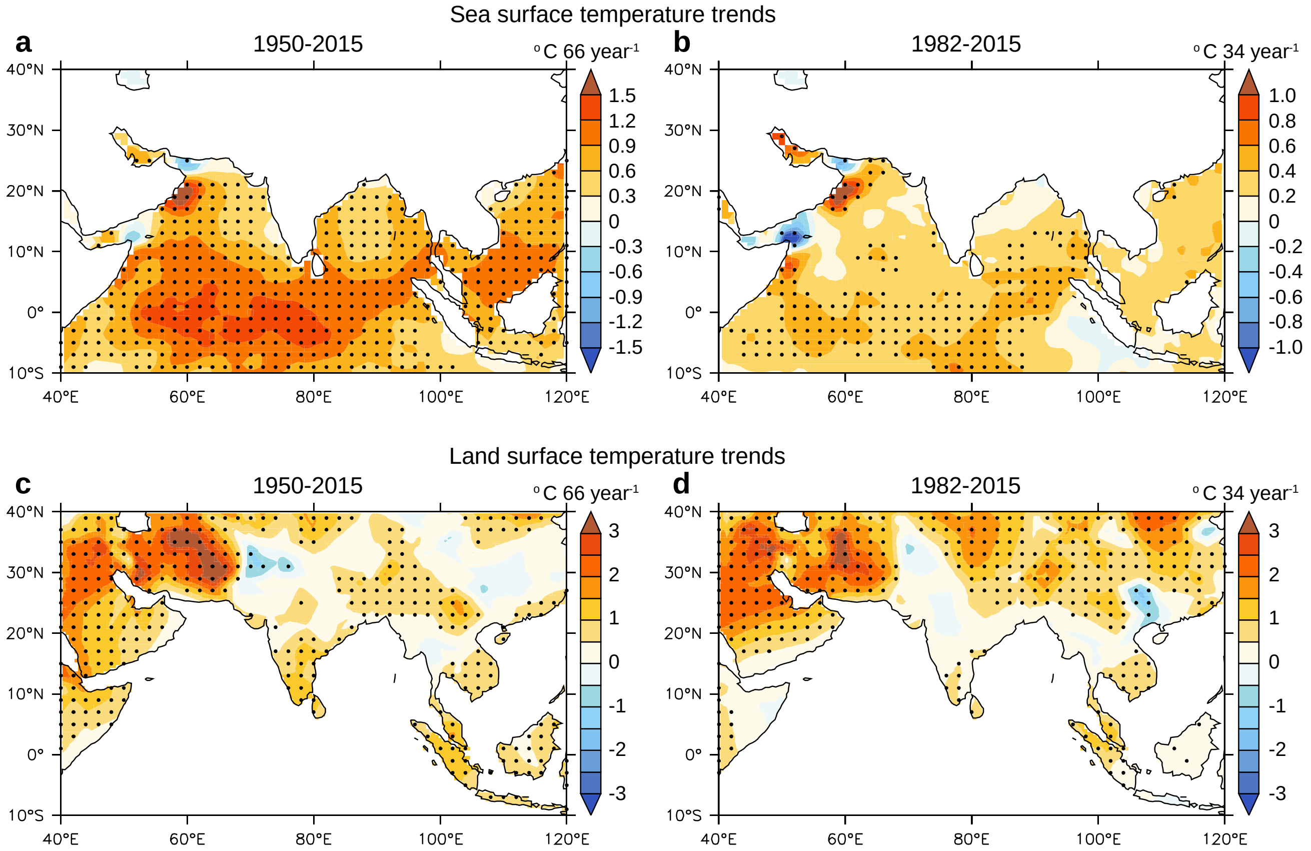

Supplementary Figure 5. Trends in the sea and land surface temperature trends (colors, °C) during the years (**a, c**) 1950-2015 and (**b, d**) 1982-2015, based on HadISST and HadCRUT datasets. Stippling indicates trends significant at 95% confidence level.

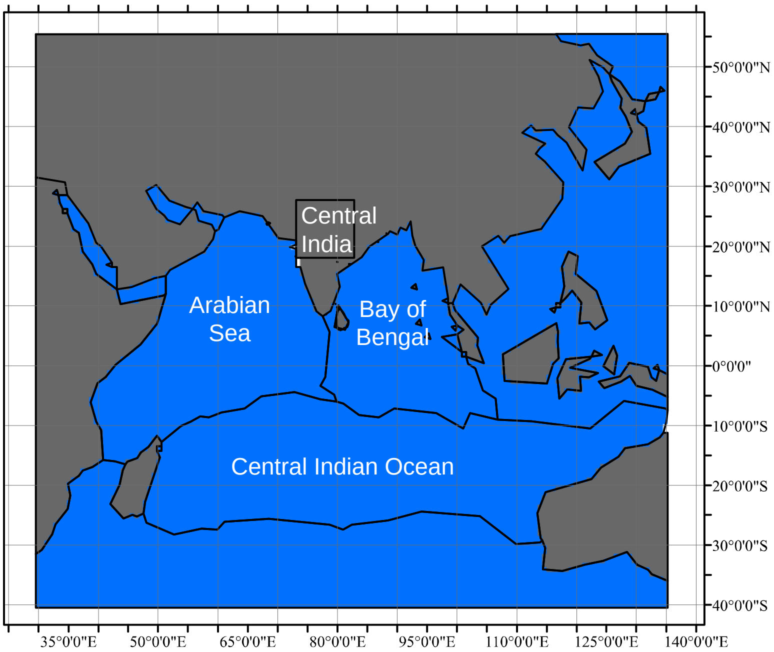

Supplementary Figure 6. Regions used in Fig. 4, for estimating the contribution from Arabian Sea (50°-75°E, 5°S-30°N), Bay of Bengal (80°-100°E, 5°S-30°N), central Indian Ocean (50°-100°E, 25°-5°S) and central Indian subcontinent (73°-83°E, 16°-28°N) in supplying moisture for the extreme precipitation events over central Indian subcontinent.

Mann-Kendall test for trends in moisture contribution from different sources			
**Moisture source**	**Significance**	**P value**	**Theil Sen's slope**
Arabian Sea	Y	0.04	0.06
Bay of Bengal	N	0.08	0.04
Equatorial Indian Ocean	N	0.22	0.01
Central India	N	0.16	0.02

Supplementary Table 1. Mann-Kendall test for trends in moisture contribution from different sources. Only the Arabian Sea contribution is showing a positive significant trend (95% confidence levels), while the other regions do not show any significant trend.

Supplementary References

1. Dominguez, F., P. Kumar, X. Liang, and M. Ting, 2006: Impact of atmospheric moisture storage on precipitation recycling. *J. Climate*, 19, 1513–1530, doi:10.1175/JCLI3691.1.
2. Dominguez F. and P. Kumar, 2008: Precipitation Recycling Variability and Ecoclimatological Stability—A Study Using NARR Data. Part I: Central U.S. Plains Ecoregion. *J. Climate*, 21, 5165–5186. doi:10.1175/2008JCLI1756.1.
3. Martinez, J. A., and F Dominguez, 2014: Sources of Atmospheric Moisture for the La Plata River Basin. *J. Climate*, 27, 6737–6753.doi:10.1175/JCLI–D–14–00022.1.
4. Pathak, A., S. Ghosh, and P. Kumar, 2014: Precipitation Recycling in the Indian Subcontinent during Summer Monsoon. *J. Hydrometeor*, 15, 2050–2066.doi: 10.1175/JHM-D-13-0172.1.
5. Pathak, A., S. Ghosh, J. A. Martinez, F. Dominguez and P Kumar, 2016: Role of Oceanic and Land Moisture Sources and Transport in Seasonal and Interannual Variability of Summer Monsoon in India, *J. Climate*, doi:10.1175/JCLI-D-16-0156.1.
